# Towards healthy One Planet cities and communities: planetary health promotion at the local level

**DOI:** 10.1093/heapro/daab120

**Published:** 2021-12-12

**Authors:** Trevor Hancock, the IUHPE’s Global Working Group on Waiora Planetary Health

**Affiliations:** School of Public Health ad Social Policy, University of Victoria, 494 Ker Ave, Victoria, BC V9A 2B7, Canada

**Keywords:** planetary health, healthy cities, Anthropocene, One Planet living, ecological determinants

## Abstract

Health promotion has paid a lot of attention to the social determinants of health and to health equity but much less attention to the ecological determinants. Yet the most fundamental determinants of health are the natural systems that make the Earth liveable and are the source of our air, water, food, fuels and materials. Yet they are threatened by the very economic and social development that we have created to meet the social determinants of health. Moreover, the benefits and burdens of that development are inequitably distributed, resulting in both ecological and social injustice. In the past few years the new field of planetary health—‘the health of human civilization and the state of the natural systems on which it depends’—has emerged, while WHO has confirmed that ‘the source of human health [is] nature’. So arguably the most important task facing health promotion in the 21st century is to turn its attention to planetary health: health promotion workers must become planetary health promoters. Local health promotion in the 21st century needs to incorporate the concept of planetary health promotion and its application in the creation of healthy ‘One Planet’ communities and must become part of the emerging network of community organizations and individuals working to create sustainable, just and healthy communities.

**Text Box**

**Links to examples of healthy and sustainable/One Planet community initiatives**

**People-Planet-Health project**

https://www.people-planet-health.com/

Inspired by the IUHPE Rorotua Statement and the UN’s Sustainable Development Goals, this project seeks to capture and celebrate the processes and actions of locally led organizations all over the world that are making valuable contributions towards building a healthy, sustainable future. Examples can be found at https://www.people-planet-health.com/stories

**Planetary Health Alliance**

https://www.planetaryhealthalliance.org/case-studies

*In November 2017, the Planetary Health Alliance* issued a call for planetary health case studies to strengthen and expand the field of planetary health by shining a light on cross-sectoral solutions that optimize human health in the face of anthropogenic environmental change. See e.g.

Going Circular: How restoring a river ecosystem in Chile’s capital city has benefitted human health and economics

https://www.planetaryhealthalliance.org/case-study-going-circular

*Te Whare Hauora o Te Aitanga-a-Hauiti* (Te Aitanga-a-Hauiti’s House of Wellness), also known as Hauiti Hauora was established by the people of Te Aitanga-a-Hauiti to rebuild and use traditional Māori models of wellness to address community health issues over twenty five years ago. Tribal leadership works to support the survival of the reo (language), tikanga (values) and kawa (protocols) with an emphasis on the ancestral landscape and the tribal relationship to the environment.

https://www.facebook.com/Te-Whare-Hauora-o-Te-Aitanga-a-Hauiti-359370924272120

*The Vava’u Environmental Protection Association (VEPA)*

https://vavauenvironment.org/#

VEPA was started in May 2009 by a group of local leaders concerned with the pressing environmental conservation issues facing the islands of Vava’u. VEPA has four focus areas aimed at ensuring biodiversity and conservation, increasing knowledge exchange, and securing sustainable livelihoods.

*Bioregional—One Planet Living*

https://www.bioregional.com/resources/#cities-and-local-government

The One Planet Showcase at https://www.oneplanet.com/futuremakers contains dozens of organizations and examples of One Planet Action Plans, primarily from the UK, Canada, Australia, South Africa and the USA, but also from Israel, Finland, India, Zimbabwe, Chile, the Netherlands, Luxembourg and the Russian Federation.

*BC Healthy Communities*

*PlanH*, implemented by BC Healthy Communities Society, supports local government engagement and partnerships across sectors for creating healthier communities across 3 critical and interconnected themes that are required for a healthy community Healthy People, Healthy Society and Healthy Environment.

https://planh.ca/

*Healthy Natural Environments Action Guide—*This guide is for local governments across the province working to create healthy, equitable natural spaces. Community planning that improves the condition and accessibility of a natural environment positively impacts a community’s health.

http://bchealthycommunities.ca/project/healthynaturalenvironments/

## PLANETARY HEALTH PROMOTION

*‘the state of the planet is broken. Humanity is waging war on nature. This is suicidal.’*

*      Antonio Guterres, UN Secretary General, at Columbia University’s World Leaders Forum, December 2nd 2020*

In this article, we argue that the most important task facing health promotion in the 21st century is to turn its attention to planetary health: health promotion workers must become planetary health promoters. The basis for this claim is very simple and is summed up in the words above from an important December 2020 speech by the UN Secretary General, Antonio Guterres. But, he added:

‘Let’s be clear: human activities are at the root of our descent towards chaos. But that means human action can help solve it’ (Guterres, 2020).

And that, we argue here, must include action by health promoters. From its inception health promotion was intended to address ecological issues, was about empowering people and had a strong focus on local action. This triad forms the basis for suggesting that health promoters working at the local level, where most of us do work, must work with our communities to create healthy One Planet communities.

### Health promotion always was socio-ecological, empowering and with a strong local focus

The Ottawa Charter for Health Promotion (WHO, 1986) contains within it three important points that are key to the focus of this article.

- First, in its rather short list of prerequisites for health, the Ottawa Charter included ‘a stable ecosystem and sustainable resources’, the first time that the WHO recognized these ecosystem functions as determinants of health. The Charter also stated ‘The inextricable links between people and their environment constitutes the basis for a socio-ecological approach to health’ and that ‘the protection of the natural and built environments and the conservation of natural resources must be addressed in any health promotion strategy’. And in its ‘Commitment’ section, participants pledged, among other things, to ‘address the overall ecological issue of our ways of living’.

But with the advent of population health in the early 1990s, the focus shifted to the *social* determinants of health; population health was ‘largely silent on ecological issues’ (Labonté, 1995) and in effect ‘mainstream population and public health has become largely ecologically blind’ (Hancock, 2015).

- Second, the definition of health promotion in the Charter—‘the process of enabling people to increase control over and improve their health’—is about empowerment for people. But this also speaks to a certain extent to the issue of scale. While some people may be able to inspire action and effect change at a large, even global scale (think Greta Thunberg), for most people it's a matter of having more control over the more immediate, local determinants of health in the settings where they lead their lives.
- Third, the Charter introduced the concept of settings, noting ‘health is created and lived by people within the settings of their everyday life; where they learn, work, play and love’. Those settings, the Charter also noted, include ‘school, home, work and community settings’; the last of these in fact contains all the others.

So, in this article, we focus first on the ecological determinants of health and the need for health promotion in the 21st century to embrace planetary health, before turning to the importance of engaging people in local actions that bring health and sustainability together in the process of creating more healthy, just and sustainable ‘One Planet’ communities, recognizing that cities and other communities contain within them most of the other important settings noted above.

### The ecological determinants of health, planetary health and the Anthropocene

We cannot claim ignorance of the health implications of the massive and rapid global ecological changes we have created; McMichael (McMichael, 1993) warned us almost three decades ago. Our understanding of the determinants of health has expanded in recent years beyond a focus on the social determinants of health (WHO Commission on the Social Determinants of Health, 2008) to encompass the ecological determinants of health (Canadian Public Health Association, 2015) and the concept of planetary health, which the Rockefeller-Lancet Commission on Planetary Health defined as ‘the health of human civilization and the state of the natural systems on which it depends’ (Whitmee *et al.*, 2015).

The recognition of the ecological determinants of health and the concept of planetary health has helped to broaden the scope of health promotion, bringing regional and global ecosystem changes into focus. But this is not about displacing the social determinants of health; far from it. As the Ottawa Charter noted, we need a ‘socio-ecological approach to health’, or as the CPHA report on the ecological determinants of health put it, an eco-social approach, while the Commission on Planetary Health defines planetary health in a way that includes human civilization.

This fits well with the understanding within Earth System Science that the Earth ‘behaves as a single, self-regulating system comprised of physical, chemical, biological ***and human*** components’ (Earth System Science Partnership, 2001, emphasis added), a living planet—Gaia—as proposed by the planetary scientist, Lovelock (Lovelock, 1979).

The power with which humanity now disrupts and harms the Earth’s natural systems is immense—so much so that it has been proposed that we are now entering a new geological epoch, the Anthropocene (International Commission on Stratigraphy, 2019), so named not because it is the age of and for humans—*anthropos* being the ancient Greek word for human—but because it is a geological epoch created by humans. We have become a disruptive force at planetary scale, So the Anthropocene is not about us, it is because of us.

It is important to note that although it is common to refer to humanity’s impact on the Earth’s natural systems, it is not humanity as a whole that is responsible—and culpable—for this harm. Rather, it is high-income countries and people who have a disproportionate impact by taking far more than their fair share of the Earth’s bio-capacity and resources. In doing so, they deprive many people around the world of their fair share—and thus their opportunity to achieve health for all. (See next section for a further exploration of this issue.)

This is not the place to review in depth the extent of the Anthropocene or its health implications; readers are referred to the works cited here, especially CPHA (CPHA, 2015) and Whitmee *et al.* (Whitmee *et al.*, 2015), as well as to the new journal *Lancet Planetary Health* and a forthcoming chapter, ‘Gaia: The ultimate setting for health promotion’ (Hancock, 2021). But, here is a brief summary of some key points.

- *The Anthropocene is much more than just climate change, important though that is.* We have crossed or are approaching planetary boundaries that should not be transgressed in a number of key Earth systems (Steffen *et al.*, 2015a,b)
- *These massive changes have occurred very recently, in ecological or geological terms*. The Anthropocene Working Group of the International Stratigraphic Commission (2019) has proposed the mid-20th century as the start of the Anthropocene. This is only 70 years ago, within the lifetime of many people alive today.
- *Since the mid-20^th^ century*, *there has been a ‘Great Acceleration’ in the trends of both socio-economic and Earth System conditions*, with the former growing swiftly while the latter has declined equally swiftly (Steffen *et al.*, 2015a).
- *Overall, humanity’s Ecological Footprint—*the amount of biocapacity we use as resources, to extract resources and to dispose of wastes—*exceeded the biocapacity of the one and only planet we have to live on in about 1970*; it is now equivalent to 1.7 Earths annually (Global Footprint Network, 2019).
- At the same time, and really as a result of this massive and rapid increase in humanity’s appropriation of ecosystems goods and services and its impact on natural systems, *the Living Planet Index (LPI) has declined precipitately since 1970*. The LPI, which monitors almost 20 811 populations of 4392 species of land, freshwater and marine vertebrates (mammals, birds, fish, amphibians, reptiles), has *declined 68% between 1970 and 2016*, the latest date for which data is available (WWF, 2020).
- *The decline in the LPI is even more dramatic and alarming in some regions and ecosystems*. It has declined 94% in the tropical sub-regions of the Americas, while the Freshwater Living Planet Index has declined by an average of 84% (WWF, 2020).
- The Secretariat of the Convention on Biological Diversity (Secretariat of the Convention on Biological Diversity, 2020) notes ‘*Biodiversity is declining at an unprecedented rate, and the pressures driving this decline are intensifying*’, while, the chair of the Intergovernmental Science-Policy Platform on Biodiversity and Ecosystem Services (IPBES) stated:

‘The health of ecosystems on which we and all other species depend is deteriorating more rapidly than ever. *We are eroding the very foundations of our economies, livelihoods, food security, health and quality of life worldwide*’ (Watson, 2019).

- *We have triggered a sixth Great Extinction* (Ceballos *et al.*, 2020), thereby depriving many other species of their rights to even exist.

All of this—and more—was synthesized in a devastating summary by Antonio Guterres, the Secretary General of the United Nations, in his December 2020 ‘State of the Planet’ address, as noted at the beginning of this article. But he also noted:

‘Making peace with nature is the defining task of the 21st century. It must be the top, top priority for everyone, everywhere’.

And that of course includes health promotion, because the impacts of these changes on the health of people—especially the most disadvantaged and vulnerable—and the wellbeing of communities and societies are already occurring (think of severe weather events, forest fires, rising sea levels, depletion of fisheries, pollution) and will become increasingly profound.

### The health and health equity implications of the Anthropocene

It should be obvious from the previous section that we cannot exceed the carrying capacity of the Earth for very long without its natural resources, at some point, becoming exhausted and its natural systems collapsing, posing an enormous threat to health. Yet that is how we are living, especially in high-income countries. Canada, for example, has an ecological footprint per person (EFpp) equivalent to 4.7 planets (Global Footprint Network, 2019).

The health implications of the Anthropocene, while touched on in what follows, are not discussed at length here—see instead CPHA (CPHA, 2015) and Whitmee *et al.* (Whitmee *et al.*, 2015), the various relevant Lancet Commissions and *The Lancet Planetary Health*, as well as the UN Environment Programme’s *Global Environment Outlook 6*, sub-titled *Healthy Planet, Healthy People*, which reviews ‘the state of the health of the environment and the related health of the people’ and notes ‘a healthy planet is important for the health and well-being of all people’ (UN Environment, 2019).

Instead, the focus is on the implications for health equity, which are rooted in the glaringly obvious inequities in the amount of bio-capacity appropriated by different countries and the related scale of harm inflicted on the Earth by high-income countries and people compared to low-income countries and people.

- The 1.1 billion people living in high-income countries (HICs) have, on average, an EFpp equivalent to 3.7 Earths, while the 3.7 billion people—half of humanity—living in low and low-middle income countries (0.93 billion and 2.77 billion respectively) have an EFpp equivalent to 0.6 and 0.8 Earth’s worth of biocapacity (Global Footprint Network, 2019).
- The International Resource Panel (International Resource Panel, 2017) reports that global resource use more than tripled between 1970 and 2017, ‘with high-income countries consuming ten times more per person than low-income countries’.

Sadly, while the high-income countries and people reap the greatest benefits in terms of human, social and economic development, it is the low-income, vulnerable countries and populations who disproportionately bear the burden of harm. The impact of these changes on health and wellbeing is already and will continue to be inequitable. While this has long been apparent for climate change (McMichael *et al.*, 1996; Intergovernmental Panel on Climate Change, 2001; Costello *et al.*, 2009; Watts *et al.*, 2015), it is true of other aspects of the Anthropocene. For example:

- The Lancet Commission on Pollution and Health found not only that ‘Diseases caused by pollution were responsible for an estimated 9 million premature deaths in 2015 - 16% of all deaths worldwide’ (and this is ‘almost certainly’ an underestimate), but that
  ‘Pollution disproportionately kills the poor and the vulnerable. Nearly, 92% of pollution-related deaths occur in low-income and middle-income countries and, in countries at every income level, disease caused by pollution is most prevalent among minorities and the marginalized’,
  with children being particularly vulnerable (Landrigan *et al.*, 2018).
- ‘The projected decline in biodiversity will affect all people, but it will have a particularly detrimental effect on indigenous peoples and local communities, and the world’s poor and vulnerable, given their reliance on biodiversity for their wellbeing’ (Secretariat of the Convention on Biological Diversity, 2020).

Moreover, high-income countries and people also deprive future generations of their fair share, thus violating the fundamental principle of sustainable development, which is ‘development that meets the needs of the present without compromising the ability of future generations to meet their own needs’ (World Commission on Environment and Development, 1987). Or as the Commission on Planetary Health noted, in population health terms we are ‘mortgaging the health of future generations to realize economic and development gains in the present’.

### WHO and the Anthropocene

*‘Protect and preserve the source of human health: Nature … the original source of all clean air, water, and food’.*

Source: WHO 2020c

If ‘making peace with nature is the defining task of the 21st century’ (Guterres, 2020), it is one to which the WHO has begun to turn its attention in recent years. WHO notes that it is ‘committed to pursuing sustainable development in all its work to help protect the people of tomorrow from the health growing health risks of today’. Currently, WHO reports that ‘Globally, 23% of all deaths could be prevented through healthier environments’ (WHO, 2019), while a global strategy on health, environment and climate change was released in 2020 (WHO, 2020a).

The health implications of climate change began to receive serious attention in the Second Assessment Report of the Intergovernmental Panel on Climate Change (Intergovernmental Panel on Climate Change (IPCC), 1996), and WHO has been involved in addressing this issue since at least the early 21st century (WHO, 2003). In 2008, World Health Day focused on the need to protect health from the adverse effects of climate change, with the then-Director General of WHO, Dr Margaret Chan stating: ‘climate change endangers health in fundamental ways’. The first Lancet Commission on Climate Change and Health (Costello *et al.*, 2009) called climate change ‘the biggest global health threat of the 21st century’, as did WHO in 2015.

WHO currently has a significant program of work on climate change and notes that climate change

‘threatens the essential ingredients of good health - clean air, safe drinking water, nutritious food supply, and safe shelter - and has the potential to undermine decades of progress in global health’, concluding ‘the Paris Agreement on climate change is therefore potentially the strongest health agreement of this century’.

WHO has also been addressing the health implications of the broader ecological changes of the Anthropocene since at least 2005. The UN’s 2005 Millennium Ecosystem Assessment (MEA), in which WHO was involved at a high level, included a report on ‘how changes in ecosystem services influence human well-being’ (MEA, 2005a), while the MEA’s Board, in its summary statement on natural assets and human well-being, issued this

‘… stark warning. Human activity is putting such strain on the natural functions of Earth that the ability of the planet’s ecosystems to sustain future generations can no longer be taken for granted’ (MEA, 2005b).

In particular, WHO has a joint work program with the Convention on Biological Diversity which, among other things, led to a report on biodiversity and human health (Secretariat of the Convention on Biological Diversity and the World Health Organization 2015) and more recently a report on integrating biodiversity in food-based interventions to support nutrition and health (WHO, 2020b).

The health implications of a more sustainable society and a ‘green’ economy were on WHO’s radar as long ago as 2011, (WHO, 2011a,b) and were noted in the Shanghai Declaration arising out of the Ninth Global Conference on Health Promotion:

‘People’s health can no longer be separated from the health of the planet and economic growth alone does not guarantee improvement in a population’s health’ (WHO, 2017).

But the Covid pandemic has led to a focus on the need for an economic recovery that is sustainable, just and healthy. Noting on World Environment Day 2020 that ‘Nature is our Greatest Source of Health and Well-Being’, WHO has called for ‘a healthy and green recovery from COVID-19 that places the protection and restoration of nature central’ and published a *Manifesto for a Healthy Recovery from Covid-19* (WHO, 2020c), which offers six ‘prescriptions’ for a healthy and green recovery.

Note that the first of these prescriptions is ‘preserve the source of human health: Nature’, while other ‘prescriptions’ include ‘Invest in essential services, from water and sanitation to clean energy in healthcare facilities; Ensure a quick and healthy energy transition; Promote healthy, sustainable food systems and Build healthy, liveable cities’. The sixth prescription—‘Stop using taxpayers money to fund pollution’—calls on governments to stop subsidizing fossil fuels.

### A call for planetary health promotion

*‘The conference participants call on the global community to urgently act to promote planetary health and sustainable development for all, now and for the sake of future generations.’*

The Rotorua Statement (IUHPE, 2019)

Bradshaw *et al.* (Bradshaw *et al*., 2021) believe ‘future environmental conditions will be far more dangerous than currently believed’, and that we are underestimating the challenges we face and failing to take the necessary actions to avoid ‘a ghastly future’. The massive and rapid global ecological changes that we have created across multiple Earth systems constitute the greatest threat to the health of humanity—and to health equity—in the 21st century, right up there with nuclear war and a challenge far greater than the Covid-19 pandemic. This is an existential crisis.

Addressing this challenge will require profound changes to the dominant economic and social development model that is the cause of these problems, a point famously made by Bhutan as long ago as 1972 in its development of Gross National Happiness as a replacement for GDP (Centre for Bhutan and GNH Studies). A new economics focused on wellbeing is needed (Raworth, 2017; Hancock, 2019a); interestingly, Aotearoa New Zealand is one of the first countries to actually develop a Wellbeing budget, Also needed are new legal, political and even spiritual understandings; the breadth and depth of changes needed in the core societal values and norms that underlie these systems are profound (Hancock, 2019b).

Planetary health and the necessary ethical, social, economic and other changes must therefore be the primary focus of health promotion—‘the top, top priority’, as Mr Guterres put it—in the 21st century, at all scales from the global to the local. So it is important that the next WHO Global Conference on Health Promotion, planned for Dubai in 2021, not only build on the Ninth Global Conference in Shanghai by focusing on sustainable development (WHO, 2017), but that it takes up the topic of planetary health promotion, building on WHO’s existing work in this area and the work of the 2019 IUHPE World Conference on Health Promotion in Rotorua, Aotearoa New Zealand.

The IUHPE conference theme was ‘Waiora: Promoting Planetary Health and Sustainable Development for All’ and there was a strong focus on Indigenous approaches in health promotion (Ratima *et al.*, 2019). Importantly the resulting Rotorua Statement noted ‘It should be read alongside the Indigenous Peoples’ Statement for Planetary Health and Sustainable Development from this Conference’.

The Indigenous Peoples’ Statement called on the health promotion community and the wider global community’ to make space for and privilege Indigenous peoples’ voices and Indigenous knowledges in promoting planetary health and sustainable development for the benefit of all’. The Statement made the point that:

‘Core features of Indigenous worldviews are the interactive relationship between spiritual and material realms, intergenerational and collective orientations, that Mother Earth is a living being—a ‘person’ with whom we have special relationships that are a foundation for identity, and the interconnectedness and interdependence between all that exists, which locates humanity as part of Mother Earth’s ecosystems alongside our relations in the natural world.’

Planetary health promotion needs to take these perspectives to heart.

But for many whose lives and work are more locally based, the question is not how we change global or national systems, but what we can do in our own backyards, both in our work lives and in our lives as citizens and community members. How do we take the global concepts discussed above and apply them locally, and what does it mean for health promotion practice?

## TOWARDS HEALTHY ONE PLANET CITIES AND COMMUNITIES

*‘Make all urban and other habitats inclusive, safe, resilient, sustainable and conducive to health and wellbeing for people and the planet’.*

     *One of four key action areas highlighted in the Rotorua Statement (*IUHPE, 2019*)*

Goal 11 of the UN’s Sustainable Development Goals (SDGs) is focused on ‘Sustainable cities and communities’ and thus was a key focus of Habitat III, which resulted in the New Urban Agenda (UN Habitat, 2016). For the first time at a Habitat conference, health was an important topic and WHO played an active role (WHO and UN-Habitat, 2016), with WHO declaring health to be ‘the pulse of the New Urban Agenda’ (WHO, 2016a). And shortly thereafter, more than 100 mayors from around the world noted in the Shanghai Consensus Statement that ‘health and sustainable urban development are inextricably linked’ (WHO, 2016b).

As a result, links between health and sustainability play a prominent part in WHO’s Urban Health Initiative, with resources that include ‘Strategies for healthy and sustainable cities’ in the areas of Energy-efficient transport, Healthy urban planning, Healthy urban diets, Slum upgrading, Healthy, energy-efficient housing and Improved urban waste management, as well as a discussion of ‘Sustainable Development Goals (SDGs) in Urban Health’ and ‘Guidance and tools’ in areas such as air pollution, household energy, walking and cycling, sustainable transport and green spaces.

WHO’s European Healthy Cities initiative, as befits an approach which grew out of the Ottawa Charter, from the outset recognized the links between health and ecological sustainability—‘An ecosystem which is stable now and sustainable in the long term’ is the second parameter of a healthy city (Hancock and Duhl, 1988). A 1996 WHO Europe book was focused on *Policies and Action for Health and Sustainable Development* (Price and Tsouros, 1996) and this focus has become more prominent with time (Hancock, 1996, 2000).

Thus the Implementation Framework for Phase VII (2019–2024) of the WHO Europe initiative notes that a key feature is

‘governance for health and well-being, which serves to reinforce the vision of health and well-being at the heart of equitable and sustainable local development’,

while one of the strategic goals is to ‘To promote policies and action for health and sustainable development at the local level’.

Theme 6 focuses on ‘Protecting the planet from degradation, including through sustainable consumption and production’, with the priority issues being climate change mitigation and adaptation; protected biodiversity and transformed urban places; health-promoting and sustainable municipal policies and waste, water and sanitation (WHO Europe, 2019). Among the key publications available on its website are factsheets on transport, health and the environment; urban planning and health; a health economic assessment tool for walking and cycling, and an action brief on urban green spaces.

### Healthy One Planet communities

*‘Imagine a world where everyone, everywhere lives happy, healthy lives within the limits of the planet, leaving space for wildlife and wilderness. We call this One Planet Living, and we believe it’s achievable.’* (Bioregional – One Planet Living)

The One Planet Living approach has been championed since 2002 by the WWF and by Bioregional, a UK-based consultancy responsible, among other things, for developing BedZED, a socially and environmentally sustainable development in south London (Desai and Riddlestone, 2002; Desai *et al.*, 2006).

Bioregional’s One Planet approach is guided by a set of 10 principles:

- Health and happiness
- Equity and local economy
- Culture and community
- Land and nature
- Sustainable water
- Local and sustainable food
- Travel and transport
- Materials and products
- Zero waste
- Zero carbon energy (Bioregional, 2015).

This list is notably different from many of the more standard ‘sustainable community’ principles because the first three principles are not about the environment at all but are about people and the community, with health and happiness being the first principle, followed by equity and local economy, and then community and culture.

Given its focus on focus on ‘happy, healthy lives within the limits of the planet’ and the prominence of health and happiness as the first principle, there is a clear relationship between the Healthy City/Community approach and One Planet Living, which suggests the need to integrate the two concepts (Hancock *et al.*, 2017; Hancock, 2018).

But the challenge of rapidly reducing our ecological footprint to the equivalent of one planet’s worth of bio-capacity is considerable. Globally, it requires an overall reduction of our footprint by 42%, but high-income countries, with an average footprint of 3.7 Earth’s (Global Footprint Network, 2019), need to reduce their footprints by 73%, so they are taking only their fair share of the Earth’s biocapacity.

Since cities produce about 80% of global GDP (UN Habitat, 2016) and are responsible for more than 75% of natural resource consumption, 60–80% of energy consumption and 75% of global carbon emissions (Steiner, 2013), much of this reduction has to occur in cities.

But the issue of equity as a One Planet principle is important here, with dramatic ecological, social and health inequity not only between rich and poor countries and cities but within cities. Most glaringly, it is important to note that there are ‘a staggering 2 to 3 billion people—35–50% of the urban population in 2050—expected to be living in informal settlements’ (UN Environment, 2019, p. 84). Meeting their social needs within the limits of one planet will be paramount; they need more, not fewer resources.

The health promotion challenge in high-income countries, then, is how do we maintain and indeed even improve health and wellbeing while at the same time dramatically and rapidly reducing the ecological footprint of our communities—and how do we make this transition to a more healthy and sustainable future in a way that is socially and economically just?

Conversely, the global challenge is to increase the share of the world’s biocapacity and resources for low-income countries and cities so they can get their fair share, which is going to require global and local redistribution (see e.g. Raworth, 2017).

### The health co-benefits of a One Planet community

The leading role of health and happiness, equity and community means that the One Planet approach is particularly suited for local health promotion action. Indeed, while a full health impact assessment of Bioregional’s flagship BedZED project (where the founders of Bioregional themselves live—talk about proof of concept!) has not been conducted, the assessments that have been undertaken show environmental, social and health benefits (see the case study in Hancock, Desai and Patrick, 2020).

Fortunately, there are many health co-benefits of a more sustainable, One Planet way of life and some of these are noted here; they are all areas in which health promotion and public health workers are already active in many communities.

1. *A low/net zero carbon energy system* not only results in large health co-benefits due to reduced global warming but the reduced air pollution results in reductions in respiratory, cardiovascular and other diseases (Smith *et al.*, 2013).
2. *Active transportation and public transit* (and a ‘smart growth’ or New Urbanist urban form that supports such a system) results in a wide range of health co-benefits including reduced greenhouse gases, air pollution and motor vehicle crash deaths and injuries, increased physical activity and reduced obesity, as well as mental and social health benefits. (Frumkin *et al.*, 2004; Sallis *et al.*, 2016).
3. *An ecologically sustainable low-meat diet*, as proposed by the EAT Lancet Commission on a healthy and sustainable diet, ‘confers both improved health and environmental benefits’ In fact, the authors estimate that the diet they propose would save 11 million lives annually, worldwide (Willett *et al.*, 2019). Interestingly, the new Canada Food Guide, which was developed solely on health grounds, recommends a very similar diet to that recommended by the EAT Lancet Commissions. While agri-food systems are not strictly speaking a local or community issue, many of the proposals for changing to this more healthy and sustainable diet focus on the importance of local food systems, including local food policy councils, community gardens and community-based agriculture (see e.g. Harper *et al.*, 2009).
4. *Increased ‘greening’ of communities*, enabling greater contact with nature, results in many mental and social wellbeing benefits (Kuo, 2015; WHO Europe, 2016).

This is by no means an exhaustive list, but these are areas in which large health and environmental co-benefits can be expected, and where much activity is already underway in many communities around the world. Other areas of importance include conservation and restoration of natural habitat; management, control and elimination of toxic materials; reduction in consumption of ‘stuff’ and a move to zero waste, and protection of watersheds and water quality. All of these changes will benefit both the environment and the health of the community, making them legitimate and important areas of work for planetary health promoters.

### Examples of local action for healthy One Planet Communities

There is not space here to include a set of case studies and examples, and anyway, it is early days yet. While there are many examples of both Healthy Community and Sustainable Community initiatives dating back decades, and a growing number of ‘One Planet’ community initiatives, there are fewer examples of integrated healthy and sustainable community initiatives or healthy One Planet initiatives. Links to some examples are provided in the Text Box.

## CONCLUSION

We live not only in the Anthropocene, but in an urban age, with more than half the world’s population living in cities, a share that is expected to reach 60% by 2030 and two-thirds by 2050 (UN Environment, 2019, p. 31). Moreover, social injustice and health inequity are very apparent in the city. So the work of health promotion, globally and locally, must be to adopt an eco-social approach (Hancock, 2015) and to achieve the principles of ‘Doughnut Economics’, meeting the social foundation for all while staying within the Earth’s ‘ecological ceiling’ (Raworth, 2017).

This will mean very different things in high, middle and low-income countries and cities. In HICs there needs to be a large and rapid reduction in the ecological footprint, while for LICs it means redistributing wealth, power and technology from HICs to enable the LICs to meet the social foundation for all their population in an ecologically sustainable manner. Health promotion must be involved in and supportive of this process in all countries, as this is a key to ensuring health for all now and for future generations.

At a local level, health promoters must integrate the local ecological and social determinants of health, identifying and emphasizing the many health co-benefits of ‘One Planet’ communities.

## DISCLAIMER

The authors alone are responsible for the views expressed in this article and they do not necessarily represent the views, decisions or policies of the institutions with which they are affiliated.
